# Cardiovascular disease and air pollution in Scotland: no association or insufficient data and study design?

**DOI:** 10.1186/1471-2458-12-227

**Published:** 2012-03-22

**Authors:** Lorna J Willocks, Abita Bhaskar, Colin N Ramsay, Duncan Lee, David H Brewster, Colin M Fischbacher, James Chalmers, George Morris, E Marian Scott

**Affiliations:** 1 NHS Lothian, Edinburgh, UK; 2 MRC Social and Public Health Sciences Unit, Glasgow, UK; 3 Health Protection Scotland, Glasgow, UK; 4 School of Mathematics and Statistics, University of Glasgow, Glasgow, UK; 5 Information Services Division, NHS National Services Scotland, Edinburgh, UK; 6 NHS Health Scotland, Glasgow, UK

## Abstract

**Background:**

Coronary heart disease and stroke are leading causes of mortality and ill health in Scotland, and clear associations have been found in previous studies between air pollution and cardiovascular disease. This study aimed to use routinely available data to examine whether there is any evidence of an association between short-term exposure to particulate matter (measured as PM_10_, particles less than 10 micrograms per cubic metre) and hospital admissions due to cardiovascular disease, in the two largest cities in Scotland during the years 2000 to 2006.

**Methods:**

The study utilised an ecological time series design, and the analysis was based on overdispersed Poisson log-linear models.

**Results:**

No consistent associations were found between PM_10_ concentrations and cardiovascular hospital admissions in either of the cities studied, as all of the estimated relative risks were close to one, and all but one of the associated 95% confidence intervals contained the null risk of one.

**Conclusions:**

This study suggests that in small cities, where air quality is relatively good, then either PM_10_ concentrations have no effect on cardiovascular ill health, or that the routinely available data and the corresponding study design are not sufficient to detect an association.

## Background

The detrimental effects of air pollution on human health came to public prominence in the mid 1900s, as a result of high pollution episodes in the Meuse valley, Belgium (1930), Donora, Pennsylvania (1948), and London, England (1952). The latter was associated with more than 3,000 excess deaths [1], and was the catalyst for legislation such as the UK Clean Air Act in 1956. Since then, a large number of studies have investigated the health risks from air pollution, and found consistent associations between components of ambient air pollution and measures of ill health. Numerous health endpoints have been considered in such studies, with the most common being mortality [2] and morbidity [3] from respiratory [4] and cardiovascular [5] disease. A number of different pollutants have also been considered in such studies, including, carbon monoxide [6], ozone [7] and both coarse [8] and fine [3] particulate matter. These studies have investigated the effects of both long-term [9] and [10] and short-term [11] and [12] pollution exposure, with the latter typically utilising an ecological time series design. Such time series studies make use of routinely available health and pollution data, and are thus the most common study design because they are inexpensive to conduct.

Studies such as those highlighted above provide evidence for the creation of air pollution legislation and guidelines, with examples in the UK being the Clean Air Act (1993) and the Air Quality Strategy (2007). In recent times, the conduit for such studies to effect UK legislation has been the independent Committee On the Medical Effects of Air Pollution (COMEAP), which informs governmental policy by critiquing the evidence provided by such studies. However, surprisingly few of these studies quantify the effects of pollution in the UK, with the majority being based in the USA [12] and [13], mainland Europe [11] and [14] or Asia [15] and [16]. As a result, COMEAP has been forced to base its recommendations to government on studies from further afield, particularly the USA. The transportation of evidence from one continent to another has its own problems (as acknowledged by COMEAP), and suggests that more studies are required to further assess the effects of air pollution in the UK. Furthermore, the studies that have been conducted in the UK have been predominantly based in London [17] and [18], with relatively few assessing the health impact of air pollution in Wales, Northern Ireland or Scotland.

In Scotland, studies estimating the short-term effects of pollution are limited to [19] and [20]. The short-term study [19] was based in Edinburgh, and investigated the effects of multiple pollutants, including nitrogen dioxide, ozone and particulate matter, on multiple health end-points between 1981 and 1995. The results from this study were largely negative, consistently showing no relationship between pollution and ill health. Study [20] extended study [19] to Aberdeen, Edinburgh and Glasgow, the three largest cities in Scotland, and performed a meta-analysis to give overall results for urban Scotland. Their study was based on data from 1981 to 2001, and also failed to find any consistent associations.

Therefore the aim of this paper is two-fold. Firstly, we extend [19] and [20] using more recent data (2000 to 2006), allowing us to add to the limited body of evidence about the effects of air pollution on health in Scotland, as well as in the UK more generally. Secondly, we provide a critique of the challenges inherent in conducting ecological time series studies of this type, particularly the appropriateness of using routinely available health and pollution data for this purpose in small to medium sized cities. Our study focuses on coronary heart disease (CHD) and stroke as the health outcomes, because they are leading causes of death and ill health in Scotland, and are clinical and public health priorities for NHS Scotland. The pollutant we consider is coarse particulate matter (PM_10_, particles less than 10 micrograms per cubic metre), because it is the pollutant most often linked to health, and because measurements of finer particulate matter (PM_2.5_ and PM_1_) are not available.

## Methods

### Data

In this study we focus on the two largest cities in Scotland, Edinburgh and Glasgow, which have populations of around 486,000 and 593,000 respectively. Daily counts of the numbers of acute hospital admissions with a primary diagnosis of CHD (ICD 10 codes I20-I25) or stroke (ICD 10 codes I61, I63 and I64) were obtained for both cities, for the seven year period spanning 2000 to 2006. This dataset (aggregated and non-disclosive) was requested from the Information Services Division (ISD) of the National Health Service (NHS) in Scotland, who provided it for the purposes of this study. Daily mean PM_10_ concentrations are measured by a network of monitors located throughout Scotland, and the corresponding data were downloaded from the Scottish Air Quality website (http://www.scottishairquality.co.uk). These data comprise measurements from three centrally sited monitors in Edinburgh and six from Glasgow, although only one and two of these monitors respectively have measurements for the entire time period (the rest did not start measuring until 2005).

An important confounding variable in existing studies of this type is temperature, and daily maximum temperatures (in Celsius) were obtained from five centrally sited monitors (three in Edinburgh and two in Glasgow) from the British Atmospheric Data Centre. In addition, daily minimum temperatures were also available at the same sites, but the results from the statistical models were invariant to choice of minimum or maximum temperature. In common with the air pollution data, the average value across all sites within a city was calculated to produce a single representative measure for each day. Finally, as our research uses routinely collected data at the ecological (population) rather than the individual level, it conforms to the Helsinki Declaration, and does not require approval from an ethics committee.

### Statistical methods

The statistical methods we adopt are similar to those used in the National Morbidity Mortality and Air Pollution Study (NMMAPS), [21] and the Air Pollution and Health a European Approach 2 study (APHEA2), [22], which are large multi-city studies based in the USA [21] and Europe [22] respectively. The daily admissions counts in each city are modelled by overdispersed log-linear Poisson models, which do not make the restrictive assumption that the mean and variance of each observation is the same. Instead, the variance is assumed to be proportional to the mean, because overdispersion (having a variance greater than the mean) is commonly seen in health count data of this type. The covariates are linked to the expected value of the response by a natural log link function, and the models were fitted using maximum likelihood methods in the statistical package R.

## Results

### Data

**Figure 1:**
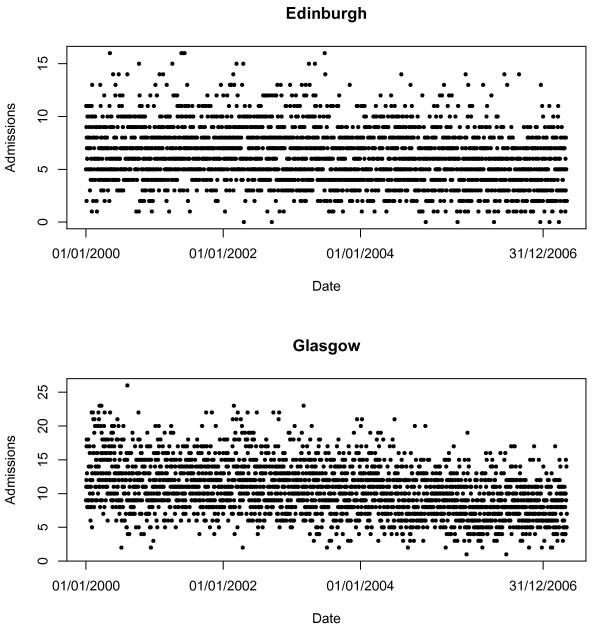
Daily numbers of cardiovascular (CHD and stroke) admissions to hospital for Edinburgh and Glasgow between 2000 and 2006.

**Figure 2:**
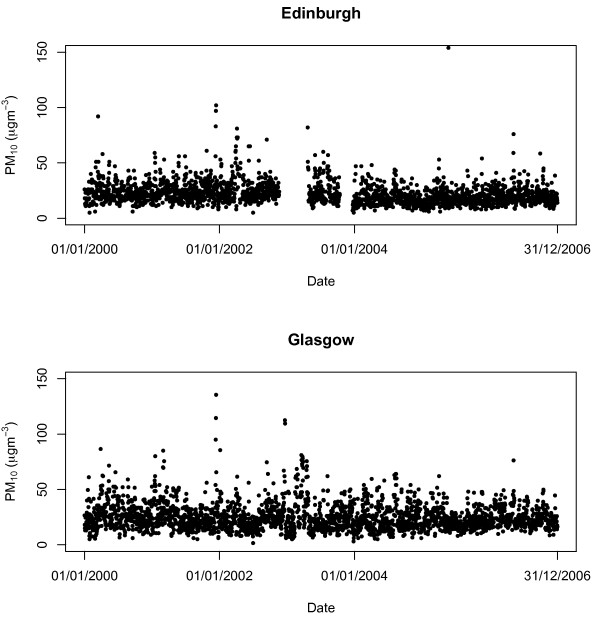
**Daily mean PM**_**10**_**concentrations for Edinburgh and Glasgow between 2000 and 2006.**

The daily hospital admissions data are displayed in Figure 1, which shows decreasing numbers of admissions in both Edinburgh and Glasgow over the seven year period. The figure also shows that the numbers of admissions per day are relatively low, with median values of 6 (Edinburgh) and 10 (Glasgow) respectively. Daily mean PM_10_ concentrations (obtained by averaging over all available measurements) are displayed in Figure 2, and show no particular trend or seasonal pattern over the seven-year period. The figure shows that the median concentrations are 20.0 (Edinburgh) and 22.5 (Glasgow) respectively, while the inter-quartile ranges are 10.0 (Edinburgh) and 13.5 (Glasgow). These concentrations compare favourably to the targets set by the UK air quality strategy, which states that by 31^st^ December 2004 the daily mean should not exceed 50 *μ*g/m^3^ more than 35 times a year. The concentrations in the two cities exceed this threshold on average only 5.7 (Edinburgh) and 13 (Glasgow) times a year over the seven-year period, which suggests that air quality in urban Scotland is relatively good. However, Figure 2 also illustrates a limitation of using routinely available data from pollution monitors, namely that on 12.9% (Edinburgh) and 1.1% (Glasgow) of days no measurements were available. As a result, this study only used data for days where at least one pollution measurement was available. Finally, daily maximum temperatures in Edinburgh and Glasgow exhibit a pronounced yearly cycle (not shown) as expected, with inter-quartile ranges (in Celsius) of 8.2 to 16.0 for Edinburgh and 8.1 to 15.7 for Glasgow.

### Statistical modelling

The covariates used to model the daily admissions counts included daily mean PM_10_, daily maximum temperature, a day of the week effect, and a non-linear time trend. The pollution data were lagged by *k* days relative to the admissions data, to ensure that the exposure occurred before the response. Lags of between zero and five days are typically considered in studies of this type, and the results for these lags are presented in the next section. Both linear and quadratic relationships were considered between the response variable and the maximum temperature covariate, because previous studies have shown evidence of a U-shaped relationship, with increased risks being observed on very cold and very hot days. However, a linear relationship is appropriate here for both cities, which is likely to be because Scotland rarely has days hot enough to cause any adverse health effects.

Finally, we included a non-linear seasonal time trend modelled by a natural cubic spline, because this was the approach adopted in the NMMAPS [21] and APHEA2 [22] studies. The smoothness of this estimated trend is controlled by its degrees of freedom, and a value of 21 is used for both cities because it is the smallest value that removed all the trend and short-term correlation from the residuals. The presence or absence of the latter was assessed by examining the residual autocorrelation and partial autocorrelation functions, which suggested that the assumption of independence was valid. Finally, the estimated overdispersion parameters from the log-linear Poisson models were 1.01 (Edinburgh) and 0.98 (Glasgow) respectively, suggesting that for these data the Poisson assumption of equal means and variances was adequate.

### Pollution-health effects

The estimated relationships between PM_10_ concentrations and cardiovascular hospital admissions are displayed in Table 1, which presents the results for both cities at lags between zero and five days. All the results are presented on the relative risk (RR) scale, for a 10 *μ*g/m^3^ increase in PM_10_ concentrations, which is a common measure used in existing studies. The table shows that all of the estimated relative risks are close to one, with only the estimate at lag 4 in Glasgow being statistically significant (its 95% confidence interval does not contain the null risk of one). These results suggest that there is no evidence of a consistent association between PM_10_ and cardiovascular hospital admissions for either of the two cities, as only one out of the 12 results is statistically significant. Furthermore, the one statistically significant result is likely to be a result of multiple testing, because in the absence of any association, it would be expected that by chance one out of every 20 confidence intervals calculated would not contain the null risk of one.

**Table 1:** **Relative risks (RR) and 95% confidence intervals (CI) for an increase of 10 micrograms per cubic metre in PM**_**10**_**in Edinburgh and Glasgow at lags zero to five**

		Edinburgh		Glasgow
Lag	RR	95% CI	RR	95% CI
0	0.9998	(0.9825, 1.0175)	1.0017	(0.9917, 1.0117)
1	1.0015	(0.9840, 1.0192)	1.0015	(0.9915, 1.0117)
2	0.9839	(0.9664, 1.0017)	1.0013	(0.9912 1.0116)
3	0.9961	(0.9786, 1.0139)	1.0023	(0.9920, 1.0126)
4	0.9950	(0.9776, 1.0128)	1.0117	(1.0016, 1.0219)
5	0.9976	(0.9801, 1.0154)	1.0073	(0.9971, 1.0175)

To determine the robustness of these results, a number of supplementary analyses were undertaken. For these supplementary analyses the study design was aggregated to the weekly rather than the daily scale, as the null results may have been due to the lack of variation (small numbers) in the daily hospital admissions series. Similar Poisson regression models to those described in the previous section were applied to the weekly admissions data, and the relative risks for lags of zero and one week were calculated. The estimated relative risks and 95% confidence intervals for the two cities were 1.0094 (0.987, 1.0410) (Edinburgh) and 1.0034 (0.9885, 1.0185) (Glasgow) at lag zero, and 1.0074 (0.9769, 1.0388) (Edinburgh) and 0.9885 (0.9729, 1.0043) (Glasgow) at lag one, which again suggests that PM_10_ concentrations have no effect on cardiovascular hospital admissions in Scotland. The increased widths of the confidence intervals, relative to those calculated for the daily analyses, are due to a decrease in the numbers of data points after aggregation to the weekly level (2,557 compared with 365). Finally, we also assessed the effects of PM_10_ on cardiovascular mortality, and again found no consistent significant associations.

## Discussion

The main finding of this study is that there was no association between PM_10_ concentrations and cardiovascular hospital admissions in urban Scotland between 2000 and 2006. This result is consistent with the earlier studies [19] and [20] in Scotland, but goes against the majority of the published literature (see for example [11] and [13], especially the systematic review of the existing evidence published by the COMEAP in 2006 [23]. This in turn raises the question of why no association was found, and there are a number of possible explanations.

The first is that PM_10_ concentrations may not have a detrimental effect on cardiovascular ill health in urban Scotland during the time period considered by this study. The lack of such an association could be because the level of PM_10_ was too low to have an effect on cardiovascular ill health, as the concentrations observed are well below the limits set by the UK air quality strategy in 2007. In addition, PM_10_ concentrations have fallen by around 50% in the UK between 1990 and 2006 (http://www.scotland.gov.uk/Topics/Statistics/Browse/Environment/TrendPM10), and many of the existing studies that found positive associations predate such improvements in air quality. For example, in the present study the median concentrations observed in Edinburgh and Glasgow were 20 *μ*g/m^3^ and 22.5 *μ*g/m^3^ respectively, which are substantially lower than in five of the eight cities studied in [11] during the 1990s (the average concentrations in *μ*g/m^3^ in these five cities were 28, 39, 51, 52, 56).

A second explanation is that the air pollution monitoring network in Scotland may not be sufficient to accurately characterise the spatial pattern in PM_10_ concentrations across the two cities, thus potentially leading to errors in the daily exposure estimates. Such errors may occur because the pollution network measures ambient (outdoor) concentrations from a small number of centrally sited monitors, some of which are located next to busy roads. Therefore the concentrations recorded by these monitors are unlikely to be representative of the levels of pollution to which the population are actually exposed. A number of statistical approaches have been proposed to tackle this problem, including the use of exposure simulators [24] and measurement error models [25]. However, such approaches are not a substitute for improved data collection, and different monitoring strategies may be required for air pollution in the future.

A third possibility to explain the null results found in this study is that PM_10_ is not the most appropriate exposure measure, because it contains relatively coarse particles that cannot travel deep into the lungs. In fact, recent evidence (see for example [26] and [27]) has shown that smaller particles, such as PM_2.5_, PM_1_ and ultra fine particles, are likely to be more toxic to human health, in part because they can travel further into the lungs. However, as already discussed, air pollution monitoring in Scotland was relatively sparse during the study period, and PM_2.5_ concentrations were not measured consistently until early 2008 in Edinburgh and late 2004 in Glasgow.

An alternative statistical explanation of our results may be that the study design did not provide enough statistical power to detect a pollution-health relationship. The choice of a time series design for this study was made for two reasons, comparability with existing research, and the routine availability of the required data. The latter is likely to be one of the major reasons why time series designs make up the largest proportion of air pollution and health studies, because they are fast and inexpensive to conduct. However, for this study design to have enough statistical power to detect an effect, both the exposure and the response series need to have sufficient levels of day-to-day variation. For the Scottish data analysed here this may not have been the case, as the levels of variation in both series were relatively low. The inter-quartile ranges for the daily cardiovascular disease counts ranged from 4 to 8 cases per day in Edinburgh, compared with between 8 and 13 cases per day in Glasgow. The variations in the daily PM_10_ concentrations were also relatively small, with inter-quartile ranges of between 16.0 and 26.0 in Edinburgh, compared with between 17.0 and 30.5 in Glasgow.

These low levels of day-to-day variation in both series may have led to a lack of statistical power in the study, which in turn may have contributed to the null associations observed here. This suggests that such routinely available data may not be sufficient for estimating the association between air pollution and ill health in small to medium sized cities such as Glasgow and Edinburgh, where the levels of day-to-day variation in both pollution and ill health are relatively small. Therefore, in the future alternative study designs with more statistical power may be required to accurately estimate the effects of air pollution on human health in Scotland. One possibility would be conduct a cohort study similar to [26] and [27], although such a study would require individual level measurements of health and pollution exposure, which would be expensive to obtain. Such an individual level study would also require data on confounding factors such as smoking status, which are not required in ecological time series studies because the distribution of such covariates across the population under study is unlikely to change on a day-to-day basis.

## Conclusions

This study suggests that in small cities where air quality is relatively good, then: either there is no relationship between concentrations of PM_10_ and cardiovascular ill health; or that time series studies lack the necessary statistical power to detect an effect and hence may not be an appropriate study design in this context.

## Authors’ contributions

LW, CR, DB, CF and JC contributed to the conception and design of the study; AB performed the analyses supervised by DL and EMS, who advised on the statistical techniques; and DL and LW wrote the paper. Finally, all the authors contributed to the interpretation of the results and re-drafting of the paper, as well as reading and approving the final version.

## Competing interests

The authors declare that they have no competing interests.

## Pre-publication history

The pre-publication history for this paper can be accessed here:

http://www.biomedcentral.com/1471-2458/12/227/prepub
